# Pollinator Conservation Strategies: The Effectiveness of Agroecological Infrastructure in Agricultural Environments

**DOI:** 10.3390/insects17100987

**Published:** 2026-09-24

**Authors:** Marta Bonforte, Roberto Catania, Maria Augusta Pereira Lima, Gaetana Mazzeo

**Affiliations:** 1 Department of Agriculture, Food and Environment, University of Catania, Via Santa Sofia 100, 95123 Catania, Italy; roberto.catania@unict.it (R.C.); gaetana.mazzeo@unict.it (G.M.); 2 Departamento de Biologia Animal, Universidade Federal de Viçosa, Viçosa 36570-900, Brazil; maugusta@ufv.br

**Keywords:** ecosystem services, floral resources, habitat management, pollinators

## Abstract

**Simple Summary:**

Pollinators are essential for the reproduction of many wild plants and crops, but their populations are increasingly threatened by intensive agriculture and the loss of suitable habitats. This review examined whether areas of natural or managed vegetation within and around farmland, such as flower strips, field margins, hedgerows, grasslands and other semi-natural habitats, can help support pollinators. We reviewed 144 studies published between 2014 and 2025 and evaluated how these habitats affect pollinator abundance and diversity, as well as the factors that determine their effectiveness. Overall, these habitats generally supported more abundant and diverse pollinator communities, particularly where flowers and nesting resources were scarce. Their benefits depended strongly on the plant species present, habitat management, surrounding landscape and the time since establishment. Different habitat types also supported different pollinator groups, suggesting that maintaining a variety of habitats is more effective than relying on a single measure. However, increases in pollinator abundance and diversity did not always result in improved crop pollination or yield. These findings can help farmers and land managers design agricultural landscapes that better support pollinator conservation while maintaining productive and sustainable farming systems.

**Abstract:**

This review evaluates the role of agroecological infrastructures, including flower strips, hedgerows, grasslands, field margins, and semi-natural habitats, in supporting pollinator communities within agricultural systems. A review following PRISMA guidelines was conducted using Scopus and Web Of Science databases, covering studies published between 2014 and 2025. A total of 144 studies met the inclusion criteria. Overall, agroecological infrastructures positively affected pollinator abundance, richness, and diversity, particularly in intensive agricultural landscapes where floral and nesting resources are limited. Flower strips were especially effective in enhancing wild bee and hoverfly populations by providing continuous floral resources, although their effects were often concentrated near field margins. Hedgerows and semi-natural habitats contributed to long-term pollinator persistence by offering nesting sites, microclimatic stability, and diverse floral resources. The effectiveness of these infrastructures was strongly influenced by floristic composition, management practices, landscape context, and time since establishment, with many studies reporting stronger effects after the second or third year. However, benefits for crop yield were inconsistent and frequently crop-specific. This review highlights the importance of integrating diverse agroecological infrastructures into farming systems to promote pollinator conservation and enhance ecosystem services while identifying major research gaps, particularly in Mediterranean agroecosystems.

## 1. Introduction

Pollination contributes to the reproduction of a substantial proportion of flowering plant species and to the production of many agricultural crops worldwide. It has been estimated that approximately 87% of flowering plant species depend, at least partially, on animal pollination for reproduction [1] and about 75% of the world’s leading food crops benefit from pollinator activity [2]. These contributions generate substantial ecological and economic benefits, supporting biodiversity conservation and agricultural production [3,4]. The global economic value of pollination services has been estimated at USD 195 billion to ~USD 387 billion annually [4]. Maintaining these benefits therefore depends on conserving diverse and functionally complementary pollinator communities capable of sustaining pollination services across agricultural landscapes.

However, growing evidence suggests that many pollinator populations have declined in abundance, distribution and diversity over recent decades [5,6,7]. Although the magnitude of these declines varies among taxa and geographic regions, concerns over pollinator conservation have increased substantially [3,8]. In Europe, for example, 8.9% of bee species are considered threatened [9], while butterfly distributions have declined by approximately 11% during a period of 25 years [10].

A wide range of insect taxa contribute to pollination in both natural and agricultural ecosystems, although their relative importance varies among habitats and plant species [3,11]. Among them, bees (Hymenoptera: Apoidea), comprising more than 20,000 described species worldwide [12], represent a major group of animal pollinators and are largely dependent on floral resources, particularly on pollen for larval provisioning [13]. In Europe alone, the most recent taxonomic checklist recognises 2187 wild bee species [14]. However, other insect groups, including hoverflies (Diptera), butterflies and moths (Lepidoptera), beetles (Coleoptera) and wasps (Hymenoptera), also contribute substantially to pollination services, particularly in specific crops and natural plant communities [11,15].

Pollinator species differ markedly in their morphology, foraging behaviour and degree of ecological specialization [16], resulting in substantial variation in the breadth of floral resources they exploit. While generalist pollinators forage on a wide range of flowering plant species, specialists rely on a more restricted subset of floral resources, reflecting differences in resource use and ecological niche breadth [17]. These functional differences influence how pollinator taxa respond to habitat management practices and determine their contribution to pollination across agricultural landscapes.

Agricultural intensification is widely recognized as one of the principal drivers of pollinator decline, with increasing evidence indicating that modern agricultural practices have profoundly altered pollinator communities worldwide [18,19]. The intensification of agriculture has been accompanied by the conversion of semi-natural habitats into simplified agricultural landscapes, increasing habitat fragmentation and the widespread use of agrochemicals, all of which have contributed to the decline in pollinator populations [3,18]. These changes reduce the availability of floral and nesting resources, increase pollinator exposure to multiple environmental stressors and ultimately affect the abundance, diversity and composition of pollinator communities, potentially compromising the pollination services that sustain both natural ecosystems and agricultural production [1,3].

The growing concern over pollinator decline has stimulated considerable efforts to identify agricultural management practices capable of reconciling food production with biodiversity conservation [19]. Consequently, increasing attention has been directed towards agricultural management practices aimed at restoring habitats and reducing the pressures contributing to pollinator decline [19]. At the same time, this has motivated the interest of the European Union, which has set targets, by 2030, to reverse pollinator decline and support pollinator populations [20].

Among the different habitat enhancement measures adopted in agricultural landscapes, flower strips are one of the most widely implemented agri-environmental practices in Europe [21]. Typically established along field margins, they consist of mixtures of native flowering species designed to provide floral resources for pollinators and other beneficial arthropods [22]. In addition, these habitats can enhance ecological connectivity within agricultural landscapes by increasing the availability of resources between cropped fields and surrounding semi-natural habitats [23]. More broadly, these habitats represent a key component of agroecological infrastructures [24], comprising semi-natural or managed habitats located within or adjacent to cultivated fields, such as flower strips, grass strips, hedgerows, woodland patches, field margins and other non-cropped elements [25] that contribute to increasing landscape heterogeneity within agricultural landscapes.

Agroecological infrastructures provide a wide range of ecological functions by supplying floral resources, nesting sites, shelter and ecological connectivity for pollinators and other beneficial arthropods [3]. Consequently, they contribute not only to biodiversity conservation through the maintenance of pollinator and natural enemy communities, but also to the maintenance of ecosystem services that support agricultural production, including pollination and natural pest control [19].

However, the efficacy of agroecological infrastructures is closely linked to the availability and continuity of the resources they provide. Their effectiveness depends not simply on the presence of flower strips, hedgerows, grasslands or woodland patches, but also on their intrinsic ecological quality [25], which determines their capacity to support functionally important groups, particularly pollinators and natural enemies. Factors such as floristic composition, vegetation structure, management practices and the surrounding landscape context strongly influence the ecological quality of these habitats and, consequently, their effectiveness in supporting pollinator communities and associated ecosystem services [25]. Previous reviews have mainly focused on flower strips established specifically to support pollinator communities within agricultural landscapes [21,26] and have consistently highlighted positive effects on the abundance and diversity of pollinators, emphasising the ecological value of flower-rich field margins as a means of enhancing habitat quality. However, their scope has largely been limited to a single type of habitat, whilst relatively little attention has been paid to other semi-natural habitats, additional pollinator groups and habitats consisting of spontaneous vegetation. Furthermore, the influence of factors such as floristic composition, habitat management, time since establishment and landscape context has often been examined within individual habitat types rather than across different habitat enhancement strategies.

Most studies have been conducted in annual cropping systems, whereas perennial tree crops have received comparatively less attention, despite offering substantial opportunities for the establishment of semi-natural habitats. Furthermore, although the Mediterranean Basin is recognised as one of the world’s major biodiversity hotspots [27,28], studies assessing the effectiveness of habitat enhancement measures in Mediterranean agroecosystems remain relatively scarce. As a result, our understanding of how different habitat enhancement measures perform in Mediterranean agricultural systems and perennial tree cropping systems remains limited.

Therefore, several important knowledge gaps remain. Addressing these gaps is essential for developing evidence-based management strategies that effectively support pollinator conservation while promoting resilient and sustainable agricultural systems.

Here, we critically reviewed the available scientific evidence on the role of agroecological infrastructures, including flower strips, hedgerows, field margins, grasslands, semi-natural habitats and woodland patches, in supporting pollinator communities and the ecosystem services associated with pollinator communities. Specifically, we evaluated how these practices influence pollinator abundance, richness, diversity and community composition, while assessing the importance of floristic composition, time since establishment, management practices and landscape context in determining their effectiveness. We also examined their effects on pollinator foraging and nesting. Finally, we discussed the economic implications of implementing these habitats, including their contribution to crop pollination, ecosystem service provision and the long-term sustainability of agricultural systems.

## 2. Methods

The review was conducted in accordance with the PRISMA guidelines, and the study selection process is summarized in Figure S1 [29].

To identify relevant studies on the effects of agroecological infrastructures on crops and pollinators, we conducted a literature search using the Scopus and Web Of Science Core Collection (WOS) databases (accessed on 1 July 2026). The search strategy was designed to retrieve studies investigating the principal habitat enhancement measures implemented within agricultural systems, while capturing the major pollinator groups and concepts related to habitat management and ecosystem services. Search terms were selected to maximise the retrieval of studies directly relevant to the objectives of the review while minimising the inclusion of literature unrelated to habitat enhancement practices.

The following search query was applied using the appropriate database-specific syntax: ((“flower strip*” OR “wildflower strip*” OR “field margin*” OR “flower-rich margin*” OR “sown margin*” OR “hedgerow*” OR “flower margin*” OR “grass strip*”) AND (“pollinator*” OR “bee*” OR “bumble bee*” OR “bumblebee*” OR “hoverfl*” OR “butterfl*” OR “lepidopter*” OR “hymenopter*” OR “wasp*” OR “beetle*” OR “coleopter*”) AND (“planting*” OR “habitat enhancement*” OR “biodiversity enhancement*” OR “habitat management*” OR “sustainable agriculture*” OR “agricultural landscape*” OR “ecosystem service*”)) This search initially yielded a total of 848 documents in Scopus and 1101 documents in Web Of Science. A series of inclusion and exclusion criteria were then applied to refine the dataset and ensure the relevance of the selected studies.

To provide an up-to-date synthesis of the available evidence, only studies published between 2014 and 2025 and written in English were considered. Publications not reporting original empirical data, including book chapters, conference papers, editorials and notes, were excluded during the screening process. In addition, the selection was limited to studies conducted explicitly in agricultural settings, excluding research carried out in urban, peri-urban, or natural ecosystems without crops, in order to focus on habitat applications within managed crop production systems. Duplicate records were removed using Rayyan [30].

Titles and abstracts were initially screened to exclude clearly irrelevant records. The remaining studies underwent full-text assessment according to predefined eligibility criteria. For each eligible study, information was extracted on habitat type, crop system, geographical location, pollinator group, study design and the main pollinator-related outcomes, including abundance, richness, diversity, community composition, foraging behaviour and nesting, where available. Studies also reporting the economic implications of habitat enhancement measures were considered when relevant to the objectives of the review. After full-text assessment and application of the eligibility criteria, 144 studies were ultimately included in the review.

## 3. Results

The selected studies are summarized in Table S1, which provides an overview of their main characteristics and principal findings.

Overall, the reviewed literature showed heterogeneous responses to agroecological infrastructures, with the direction and magnitude of the reported effects varying among infrastructure types, pollinator groups, response variables, crop systems and landscape contexts. The geographical and crop-specific distribution of the selected studies is presented in Figure 1.

Germany (*n* = 19) and the United States (*n* = 26) accounted for the largest number of publications across the different crop categories included in this review, whereas studies from other countries were less frequent and generally restricted to specific agricultural systems.

Figure 2 shows the geographical distribution of the reviewed studies across the macro-geographical regions considered in this review. The Mediterranean Basin accounted for 20.30% of the selected studies, while the remaining literature was distributed across the other geographical regions.

To provide an overall synthesis of the direction of pollinator responses to agroecological infrastructures, the effects reported in the reviewed studies were classified as positive, neutral or negative separately for each pollinator group assessed within each study. Thus, each study contributed one classification for each pollinator group assessed, and studies investigating multiple pollinator groups contributed multiple group-specific classifications. When different response variables for the same pollinator group within a study showed different directions of effect, these were retained as combined response categories (i.e., positive + neutral, positive + negative, neutral + negative, or positive + negative + neutral) (Table S2). Overall, exclusively positive responses were frequently reported for wild bees (53.4%), bumble bees (53.5%), Syrphidae (49.3%), Lepidoptera (46.2%) and honey bees (41.9%). Neutral responses were comparatively frequent for Diptera (36.8%) and Coleoptera (35.3%), while combined response categories accounted for 30.2% of responses for wild bees, 23.9% for bumble bees, 33.2% for Syrphidae, 35.9% for Lepidoptera and 29.8% for honey bees. Exclusively negative responses were generally uncommon (Table S2). When the same classifications were examined according to the main agroecological infrastructure categories, exclusively positive responses predominated across all categories, although their relative frequency varied (Table S3). They accounted for 70.8% of group-specific classifications for woody habitat enhancement, 65.5% for within-field vegetation enhancement and 61.5% for habitat enhancement schemes, compared with 47.4% for semi-natural herbaceous habitat enhancement, 43.8% for multiple strategies and 41.0% for floral resource enhancement. Floral resource enhancement showed the highest proportion of exclusively negative responses (10.7%) and a comparatively heterogeneous distribution across response categories. Combined response categories were also frequent for habitat enhancement schemes, within-field vegetation enhancement and multiple strategies, reflecting variation in the direction of different response variables assessed for the same pollinator group within individual studies.

The reviewed studies described a wide variety of agroecological infrastructures using different terminology and levels of detail. To provide a more consistent synthesis of the available evidence, the agroecological infrastructures reported in Table S1 were grouped into six main categories according to their predominant characteristics (Table 1). Studies evaluating agroecological infrastructures belonging to more than one category were classified as “Multiple strategies”. The category “Habitat enhancement schemes” includes broader approaches, such as Agri-environment schemes (AES) and Farming with Alternative Pollinators (FAP), that support the establishment or management of habitats for pollinators. For each category, representative agroecological infrastructures and the most frequently investigated response variables are summarized.

### 3.1. Agroecological Infrastructures Increase Pollinators

Agroecological infrastructure represents an effective method for promoting pollinator conservation by providing food sources and nesting habitats, especially in areas with high percentages of cultivated land. Although their effectiveness varies depending on the type of habitat, the landscape context and the pollinator community, numerous studies have highlighted positive effects on the abundance and diversity of pollinators. Among agroecological infrastructures, flower strips are the most extensively investigated and provide continuous floral resources, promoting higher bee abundance, particularly bumble bees, while increasing flower species richness and floral density [32,33]. Similar positive responses have been reported across different cropping systems, including citrus orchards [34], wheat fields [35] and mango orchards [36], where flower planting increased the abundance and diversity of bees and other pollinators despite differences in crop type and geographical context. A comparison between conventional management, conventional fields with flower strips and organic farming showed the highest local abundance and species richness of bumble bees in fields with flower strips, with intermediate values under organic management [37]. Beyond their immediate effects on flower visitors, flower strips may also contribute to longer-term population dynamics. Annual flower strips enhanced the abundance of bumble bee reproductive castes, particularly queens in the following spring, indicating positive carry-over effects on population growth [38]. The benefits of additional floral resources may also depend on their availability relative to pollinator density. Wild bee species richness increased with bloom density within wildflower strips, whereas lower wild bee abundance and richness were recorded on farms with managed honey bee hives, suggesting that floral resource availability relative to the number of foraging bees may influence their conservation value [39].

Positive responses are not restricted to flower strips but have also been documented in a wide range of agroecological infrastructures. Hedgerows with high woody plant diversity supported more bumble bees, while abundant understorey floral resources particularly benefited hoverflies, with these effects being strongest in intensively managed landscapes [40], while hedgerow gardens in avocado orchards supported greater bee species diversity and slightly higher abundance than surrounding control habitats [41]. Crop field margins supported higher wild bee abundance and species richness than perennial hayfields and natural habitats [42]. Artificial field defects established within oilseed rape fields increased the abundance and species richness of bees and butterflies compared with crop controls [43]. However, hoverflies showed no significant response to the habitat treatments in either study.

Among field-edge habitats, grass margins supported higher wild bee abundance and diversity than planted and remnant hedgerows, with both metrics positively associated with floral abundance and plant species richness [44].

Restored habitats also promoted richer pollinator communities in agricultural landscapes, particularly where floral resources were abundant and diverse. Re-established grasslands [45], native prairies [46], prairie strips [47] and green covers [48] consistently supported higher pollinator abundance and richness than conventionally managed habitats, although butterfly responses depended on habitat association, with grassland species more abundant in conservation grasslands and generalists more abundant in marginal grasslands [49]. Beyond changes in pollinator abundance and richness, prairie strips also enhanced the complexity of plant–bee interaction networks and improved body condition in several wild bee taxa, despite no significant differences in overall wild bee abundance or richness compared with field margins [50].

Pollinator responses differed among taxonomic groups and their specific ecological needs. Flower strips generally favoured hoverflies, whereas butterflies benefited more from extensively managed grasslands characterised by greater vegetation stability and lower management intensity [51]. This complementarity among agroecological infrastructures was also reported in Mediterranean vineyard agroecosystems, where different ecological infrastructure types supported distinct pollinator assemblages, with flower strips particularly benefiting pollinators through the availability of floral resources [52].

Evidence from different cropping systems indicates that increases in pollinator abundance do not necessarily translate into greater pollination within crops. In potato fields, pollinator abundance increased with floral cover in adjacent margins, but this effect did not extend into the crop interior [53] or improve crop yield and seed production in melon fields despite enhancing the abundance and richness of several wild pollinator groups [54]. Likewise, native flower strips in avocado orchards increased fly visitation and fruit production, whereas wild bees remained largely confined to the strips [55]. In contrast, adjacent wildflower strips increased pollinator visitation to strawberry flowers by approximately 25%, demonstrating that even relatively small flower plantings can increase pollinator visitation to nearby crops [56]. Evidence from different cropping systems indicates that appropriate field margin management can enhance pollination services by increasing the abundance of flower-visiting insects. This resulted in higher pod production, seed number and yield in beans, and increased seed set in buckwheat [57,58]. These findings highlight that the effectiveness of agroecological infrastructures depends not only on their ability to attract pollinators, but also on the extent to which pollinators move from these habitats into adjacent crops. The extent to which pollinators move from agroecological infrastructure into adjacent crops also varies depending on the groups of pollinators. Bumble bees, solitary bees and hoverflies showed declining visitation with increasing distance from field edges, whereas honey bees exhibited little or no spatial response [59,60]. Similarly, inter-row flower strips in almond orchards were mainly utilized by *Osmia lignaria* Say, 1837 nesting within 40 m of the strips, while also supporting a diverse assemblage of wild bees, confirming that the spatial configuration of floral resources strongly influences pollinator use of agroecological infrastructures [61]. Overall, these findings indicate that increases in pollinator abundance do not necessarily translate into enhanced crop pollination. The contribution of agroecological infrastructures to pollination services depends on species-specific foraging behaviour and the movement of pollinators from these habitats into adjacent crops.

Different agroecological infrastructures may also provide complementary resources for pollinator communities, with their effectiveness varying according to habitat characteristics and seasonal resource availability. In apple orchards, perennial flower strips and hedgerows offered complementary resources over time, with hedgerows supplying floral resources early in the season and flower strips supporting more abundant and species-rich bee communities later in the year [62]. Linear woody elements, such as hedgerows and woodland patches, also supported pollinator communities by providing floral resources, shelter and nesting sites, with particularly strong effects reported for hoverflies and mining bees [63]. This complementarity was also evident among semi-natural habitats, with hedgerows, forest edges and permanent grasslands supporting distinct wild bee communities and wooded habitats providing specific pollen resources and plant–bee interactions not recorded in grasslands [64].

The complementary role of different habitats was also evident when flower strips were compared with semi-natural habitats. Semi-natural grasslands generally supported more diverse pollinator communities and a wide range of specialist species, whereas flower strips mainly sustained abundant populations of generalist pollinators, particularly honey bees and bumble bees [65]. Likewise, semi-natural habitats supported more diverse wild bee communities and more complex plant–pollinator networks than sown flower strips, although no differences in network robustness were detected between the two habitat types [66], while in agricultural landscapes dominated by oilseed rape, wildflower strips increased the diversity and evenness of aculeate pollinator assemblages whereas semi-natural grasslands maintained higher pollinator abundance, highlighting the complementary role of both habitat types in supporting pollinator communities [67]. Rather than representing alternative conservation strategies, sown habitats and existing flower-rich patches supported complementary bee communities, with naturally established habitats providing additional floral resources for specialised species and extending resource availability throughout the season [68]. Spatial constraints further influence the provision of pollination services. In apple orchards, wild bees and hoverflies frequently remained restricted to flower-rich margins rather than moving into the crop interior [69]. However, this limited movement did not necessarily reduce pollination within crops. Adjacent flower strips and hedgerows provided floral resources throughout the season without reducing pollinator visitation to apple flowers [70]. Comparable responses were observed in other cropping systems. In courgette fields, greater wildflower species richness increased bumble bee abundance on crop flowers without reducing pollinator visitation, while also supporting higher abundance and species richness of solitary bees, particularly in landscapes with limited semi-natural habitat [71]. Nevertheless, greater proximity between crops and flower-rich habitats did not always translate into improved pollination services. On small diversified farms, reducing the distance between crops and wildflower strips did not increase bee visitation, pollination or crop yield, despite the presence of highly diverse wild bee communities [72]. Likewise, flower strips in strawberry systems enhanced pollination only in areas adjacent to field margins and did not increase pollination across the entire crop [73].

Overall, these findings suggest that agroecological infrastructures generally increase pollinator abundance and diversity, although their contribution to pollination services depends on species-specific responses and the spatial distribution of floral resources within agricultural landscapes.

### 3.2. Temporal Patterns of Agroecological Infrastructures

Among the various aspects influencing the effectiveness of agroecological infrastructures, ecological maturation, understood as the time elapsed since their establishment, plays a fundamental role. Long-term studies have shown that bee and butterfly richness may increase three- to fivefold after more than two years of habitat enhancement, whereas specialised bee species respond more slowly and only become significantly more abundant from the third year onwards [74]. These findings indicate that the ecological benefits of habitat enhancement may require several years to become fully established.

The transition from the initial attraction of pollinators to sustained population growth is gradual.

Flower strips containing highly attractive species may simply concentrate existing pollinators rather than support population growth, particularly for species with a single generation per year. Indeed, floral strips established in Canadian agricultural systems rapidly attracted bees and ground beetles only a few weeks after sowing, whereas syrphids remained more abundant in unmanaged margins [75]. Early responses were also observed in a Mediterranean melon system, where flower strips affected pollinator visitation rates to melon flowers during the first year after establishment, although no effects on crop productivity or quality were detected [76], whereas in sunflower fields, increased wild bee visitation and seed set emerged only in the second year after establishment [77]. However, responses continued to develop over longer time scales. Flower strips maintained high floral richness over three years while bee abundance increased progressively, with approximately twice as many bees recorded within adjacent crop fields bordered by flower strips than in control fields, while greater bee spillover into neighbouring crops was also reported [78].

Although flower strips can rapidly attract pollinators, the persistence of their effects depends on changes in floral resources and vegetation development over time. The abundance of honey bees, wild bees and hoverflies has been shown to decline after the second year, in parallel with reductions in floral abundance and flowering plant richness [79]. Differences in temporal responses were also observed among pollinator groups. Honey bees, mining bees and hoverflies declined as margins aged, whereas bumble bees, Lepidoptera and trap-nesting bees maintained stable or slightly increasing abundances, indicating that long-term management requirements vary among taxa [80]. However, temporal patterns were not always associated with reduced habitat quality during the establishment phase. For example, delaying flower strip establishment by four weeks did not reduce plant species richness or Shannon diversity, although earlier establishment resulted in greater flowering species richness during the first growing season [81]. Vegetation structure also influenced temporal responses. In Mediterranean agricultural systems, managed herbaceous margins supported higher bee abundance during the first year, whereas shrubby margins became more favourable to several bee groups during the second year [82]. Together, these studies indicate that temporal responses vary according to both pollinator taxa and the development of vegetation within agroecological infrastructures. Temporal responses also differed among pollinator groups. Comparable patterns were observed in restored grasslands, where newly established and old grasslands showed that wild bees, bumble bees and hoverflies benefited from newly established grasslands in terms of abundance and species richness, whereas butterflies were more abundant in old grasslands [83]. Consistent with this pattern, a six-year study on restored grasslands showed that newly established grasslands maintained higher wild bee richness and supported similar richness of hoverflies and butterflies compared with permanent grasslands, confirming that responses to habitat establishment vary among insect taxa [84].

Beyond changes in pollinator abundance and diversity, temporal patterns in agroecological infrastructures may also influence other ecological processes, including overwintering, nesting and population persistence. Wild bees, for instance, appear to require habitats characterised by greater temporal continuity for successful overwintering, while habitats subject to annual disturbances can act as ecological traps [85], whereas perennial wildflower strips subjected to annual mulching showed increasing wild bee and butterfly richness with age and generally higher abundance than reference field margins [86]. Habitat restoration may also promote pollinator persistence and colonisation over time [87], with maturing native hedgerows increasingly favouring floral specialists and cavity-nesting bees [88]. As habitats become established, they can also improve reproductive and nesting opportunities. It has been shown that flower strips improve the reproductive success of solitary bees by reducing foraging distances and increasing brood production [89], as well as providing nesting opportunities for ground-nesting species [90]. Together, these findings indicate that the temporal development of agroecological infrastructures influences not only pollinator abundance, but also ecological processes that contribute to the long-term persistence of pollinator populations.

The ecological benefits associated with habitat maturation do not always translate into immediate improvements in crop pollination or productivity. In UK cider apple orchards, perennial flower strips increased the visitation rate of wild insects, particularly non-*Apis* bees and flies, but this did not translate into increased fruit set [91]. Instead, fruit set was positively associated with wild insect richness and *Andrena* visitation, whereas pollinator richness remained higher near semi-natural habitats [91]. Comparable responses were reported in sweet cherry orchards, where wildflower strips increased visitor density, richness and diversity after anthesis, particularly in managed strips, although visitor density was not directly related to fruit set [92]. Nevertheless, pollinating insects were essential for fruit production, and pollination deficits were lower in orchards containing wildflower strips than in the control treatment.

The effectiveness of agroecological infrastructures over time is also influenced by habitat quality and resource availability. In apple orchards, flower strips specifically designed for pollinators and multifunctional mixtures both supported higher pollinator richness than control or open-nectar treatments and attracted pollinators alongside other beneficial arthropods [93]. Habitat quality also depended on the availability of resources beyond floral forage. Agricultural field margins supported bumble bee communities comparable to those occurring in semi-natural habitats, while bumble bee abundance and diversity increased with floral abundance, floral diversity, rodent holes and sandy soil texture [23]. Evidence from long-term experiments further suggests that these ecological benefits can be maintained without compromising agricultural production. In a farm-scale experiment conducted over six years, the conversion of 3–8% of arable land into wildlife habitats, including grass margins, wildflower patches and margins rich in pollen and nectar resources, maintained overall crop yield despite the reduction in cultivated area and increased faba bean yield [94].

Overall, these findings indicate that the long-term effectiveness of agroecological infrastructures depends not only on the time elapsed since their establishment, but also on the development of habitat conditions capable of supporting pollinators and associated ecosystem services.

### 3.3. Selection of Plant Species

Flower composition and flowering phenology play a central role in the design of agroecological infrastructures. Selecting annual, biennial and perennial species can increase the availability of floral resources within intensively cultivated landscapes while ensuring a more continuous supply of nectar throughout the season, particularly during periods of resource scarcity [95]. Accordingly, flower plantings designed to provide a succession of flowering species promoted higher pollinator abundance and wild bee richness than non-enhanced areas [96]. Likewise, diverse seed mixtures with complementary flowering phenologies enhanced pollinator abundance while maintaining floral resources throughout the growing season, highlighting the importance of species selection during the establishment of flower plantings [97,98].

The seasonal availability of floral resources also depends on surrounding vegetation. In buckwheat, the abundance of insects visiting crop flowers increased with the richness of co-flowering wild plants during autumn, whereas no relationship was observed during summer, indicating that the contribution of surrounding floral resources may vary according to seasonal flowering dynamics [99]. Comparable patterns were reported in a deciduous fruit orchard, where greater richness of flowering weeds and higher blooming cover were associated with increased flower visitor abundance, while the richness and blooming cover of hedgerow species also influenced plant–pollinator network structure [100]. Likewise, pollinator communities were more strongly associated with vegetation composition and flower richness than with habitat type alone, highlighting the importance of local habitat quality in determining pollinator occurrence [101,102]. Similarly, the cover and species richness of wild flowering plants growing in hedgerows were positively associated with the abundance of several flower-visiting insect groups, highlighting the importance of spontaneous floral resources alongside those provided by crop fields [103]. The effectiveness of sown flower mixtures also depends on their successful establishment. Under weedy conditions, higher seeding densities, particularly in mixtures combining grasses and flowering forbs, increased the establishment of sown species, promoted a greater abundance of flowering plants and consequently enhanced bee abundance [104].

The composition of seed mixtures is equally important, as increasing plant diversity does not necessarily maximise pollinator responses. Flower strips containing *Phacelia tanacetifolia* Benth. supported a higher richness of butterfly species and attracted a greater number of specialist pollinator species than *P. tanacetifolia* monocultures, whereas bee richness and abundance did not differ between the two planting types [105]. However, highly diverse mixtures generally provide a longer flowering period. Nevertheless, honey bees and bumble bees showed peak foraging activity in monocultures and mixtures with low diversity, whereas highly diverse mixtures received lower visitation rates despite their greater temporal stability [106]. Dominant flowering species were observed when these were included in highly diverse mixtures, indicating that greater plant richness was not associated with higher pollinator visitation under the conditions of that study [106]. In Mediterranean vineyards, high-diversity sown inter-row mixtures did not provide additional benefits for wild bee or hoverfly abundance compared with spontaneous vegetation, although both vegetated treatments outperformed tilled inter-rows [107].

Different flowering plant species produced contrasting responses in pollinator behaviour and crop visitation. In strawberry, *Coriandrum sativum* L. and *Mentha arvensis* L. attracted more flower visitors than the crop itself, resulting in fewer visits to strawberry flowers, whereas *Myosotis arvensis* (L.) increased pollinator diversity without reducing crop visitation. Despite these differences, the proportion of marketable fruit did not differ among treatments [108]. Species-specific preferences were also observed in tomato field margins, where *Glebionis coronaria* (L.) Cass. ex Spach, *Anethum graveolens* L. and *Fagopyrum esculentum* Moench mainly attracted wild bees, whereas *Rapistrum rugosum* (L.) All. attracted mainly honey bees. However, the number of wild bees visiting tomato flowers was not affected by the distance from the field margin, indicating no evidence of pollinator distraction under the conditions of the study [109]. Changes in pollinator distribution were also reported in faba bean, where annual flower strips modified the spatial distribution of bumble bees by increasing legitimate flower visits close to the strips, although they did not increase overall bumble bee densities or insect pollination benefits [110]. In contrast, supplementary flowering plants in kiwifruit orchards deterred *Osmia bicornis* (Linnaeus, 1758) and other pollinators away from the crop, reducing seed number and fruit dry matter [111].

A similar redistribution of flower visitors without consistent improvement in crop pollination was observed in avocado, where annual flower strips provided a refuge for honey bees and hoverflies and did not increase pollinator abundance on avocado flowers or fruit set [112]. Likewise, flower strips containing *Brassica hirta* Moench, *Fagopyrum esculentum* Moench and *Lobularia maritima* (L.) Desv. attracted beneficial insects but did not consistently increase cucumber yield, with significant benefits limited to plants adjacent to *L. maritima* [113].

Pollinator groups also differed in their preferences for floral resources. Bumble bees showed a clear preference for seed mixtures dominated by Fabaceae, whereas hoverflies were more strongly associated with Apiaceae [114]. Butterflies, however, showed no clear response to seed mixture type and were instead associated with local flower richness. In addition, the abundance of all flower-visiting insects, except butterflies, increased with the established cover or richness of sown flowering species. These responses were not restricted to sown flower strips. Larger or higher-quality flowers increased bumble bee abundance beyond the sown habitat, with positive effects detected in surrounding field margins across the wider landscape [115]. These benefits were strongest on farms containing larger or higher-quality flower strips, whereas responses of solitary bees remained more context-dependent. Species-specific associations were also reported in field margins, where restored margins supported higher bee abundance and diversity than non-restored margins, while shrubby and herbaceous margins differed in their bee community composition [116]. Honey bees and mining bees were positively associated with overall floral resource availability, whereas wild Apidae and Megachilidae were associated mainly with Lamiaceae occurring in shrubby margins [116]. Similar patterns were reported in vineyards, where native annual and perennial flowering mixtures increased pollinator abundance and species richness compared with areas without cover plants [117]. Responses differed among pollinator groups according to flower morphology and flowering phenology.

Not all flowering species contribute equally to supporting pollinator communities. In experimental seed mixtures, *Achillea millefolium* L., *Anthemis tinctoria* L., *Chenopodium album* L. and *P. tanacetifolia* together supported 81% of the recorded pollinator species, and the presence of these key species explained pollinator attraction better than overall plant species richness [118]. However, supporting diverse pollinator communities required more than selecting the most frequently visited flowering species. Although a small number of wildflower species supported a high richness of wild bees, additional flowering species contributed complementary bee assemblages, indicating that apparently redundant species may still play an important functional role in flower mixtures [119].

Pollinator preferences also differed among functional groups. Social bees preferentially foraged on clover plantings, whereas solitary bees were more frequently associated with wildflower plantings [120]. Likewise, native prairie mixtures and monocultures of *Silphium integrifolium* Michx. and *S. perfoliatum* L. supported the highest bee abundance and species richness, whereas *Medicago sativa* L. supported the highest abundance and richness of Lepidoptera [121]. Differences were also evident among bee conservation target groups, with rare bees, dominant wild crop-pollinating bees and managed honey bees relying on partly different sets of flowering species despite all responding positively to increasing plant species richness [122]. These findings emphasise that plant species selection influences not only pollinator abundance but also the composition of pollinator communities.

The selection of flowering species also influenced the effectiveness of habitat enhancement measures in agricultural systems. In coriander, a monospecific margin of *Diplotaxis tenuifolia* (L.) DC. attracted significantly more bees, hoverflies and beetles than a mixed margin while still increasing seed production [123]. In hazelnut orchards, native perennial species, including *A. millefolium*, *Geum macrophyllum* Willd. and *Prunella vulgaris* L., supported more abundant and diverse pollinator communities than commercially available cover crops, including *Hordeum vulgare* L., *Avena sativa* L. and *Trifolium repens* L., highlighting the value of native species for restoring pollinator communities in perennial cropping systems [124].

The practical application of plant species selection is exemplified by the Farming with Alternative Pollinators (FAP) approach, in which Marketable Habitat Enhancement Plants (MHEP) are selected to provide floral resources while maintaining economic returns for farmers [125]. Within this approach, MHEP are selected according to their attractiveness to pollinators, flowering phenology and farmers’ preferences, combining species with complementary floral traits and overlapping blooming periods to provide floral resources throughout the cropping season [126,127]. This approach has been successfully applied in several cropping systems. In faba bean, species such as flax, coriander, arugula, chia and canola were used as MHEP and shared flower visitors with the main crop while also hosting pollinator groups that did not meet their food requirements from faba bean alone [128]. Pollen analyses across faba bean and pumpkin systems further showed that MHEP contributed specific pollen resources, including coriander and canola for faba bean visitors and sunflower for pumpkin visitors, although FAP management did not significantly increase their overall dietary breadth or the number of pollen genera collected [129]. Evidence of complementary resource use was further supported by pollen analyses showing that wild bees frequently foraged on both native wildflower strips and adjacent peanut or cotton crops, indicating regular movement between these habitats throughout the growing season [130].

The importance of combining marketable flowering plants with surrounding wild vegetation was further highlighted by comparisons showing that, although FAP fields supported higher pollinator richness than conventional monocultures, 28% of the recorded pollinator species occurred exclusively on wild flowering plants, particularly thistles [131]. Across different crops and agro-climatic regions, FAP similarly increased wild pollinator abundance and richness at the whole-field level, although these benefits generally did not extend to the main crop and varied among crop types [132]. More recently, an evaluation across 31 trials in four Mediterranean agroecosystems using 27 MHEP identified several species that consistently supported higher abundance and diversity of flower visitors and natural enemies, while also being preferred by participating farmers [133]. Together, these studies demonstrate that effective plant species selection should consider not only the attractiveness of flowering species, but also complementary flowering phenologies, floral trait diversity and farmers’ preferences, thereby facilitating the practical implementation of agroecological infrastructures.

### 3.4. Evaluation of the Landscape Context

Beyond the agroecological infrastructure itself, the surrounding landscape context strongly influences pollinator communities and the effectiveness of habitat enhancement measures. In addition to plant species selection and flowering continuity, the composition of the surrounding landscape, including semi-natural habitats, grasslands, forests and uncultivated areas, contributes to shaping pollinator responses to agroecological infrastructures. Land-use intensification reduces habitat diversity and connectivity, simplifying plant–pollinator interaction networks, whereas local floral resource availability can partially mitigate these negative effects by supporting pollinator richness within agricultural landscapes [134]. However, the effectiveness of habitat enhancement measures varies according to landscape context. Restored hedgerows increased wild bee richness along field margins but did not consistently enhance pollination services in adjacent sunflower fields, indicating that the benefits of field-edge diversification depend on crop identity and landscape context [135].

However, local habitat enhancements alone do not necessarily guarantee positive responses across pollinator groups. In intensively managed landscapes, annual flower strips had limited effects on the abundance of solitary bees and wasps in the surrounding landscape [136]. The availability of suitable habitats in the surrounding landscape also influenced the effectiveness of local interventions. Wildflower areas established within landscapes containing semi-natural habitats and additional connected flowering habitats supported higher pollinator richness than isolated sites, highlighting the importance of landscape connectivity in enhancing the benefits of local habitat interventions [137]. Habitat quality further contributed to these responses. Hedgerow proximity interacted with floral diversity to enhance wild bee persistence and occupancy at more diverse sites, while colonisation was promoted by both local floral diversity and nearby hedgerows [138]. Differences in the quality of surrounding habitats were also reflected in pollinator activity, with foraging bumble bees being more abundant along hedgerow margins adjacent to roads than those bordering arable fields, associated with greater floral resource availability [139].

Different semi-natural habitats also contribute in complementary ways to sustaining wild bee communities across agricultural landscapes. Extensively managed meadows, flower strips, hedgerows and forest edges each supported distinct arthropod assemblages, indicating that no single habitat type provides all the resources required throughout the season [140,141]. A similar pattern was reported across the different habitats occurring within organic farms. Different low-input habitats, including meadows, pastures, herbaceous strips, flower strips and organic cereal fields, supported distinct wild bee assemblages, indicating that a mosaic of complementary habitats is required to maintain high wild bee diversity within agricultural landscapes [142]. Habitat complementarity also depended on the type of resources provided. Although hedgerows supply abundant floral resources for foraging bees, they do not necessarily enhance nesting habitat for ground-nesting species, suggesting that many bees use these habitats primarily for foraging while nesting elsewhere [143]. This distinction was also reflected in perennial wildflower strips, where landscape effects were stronger for solitary than social wild bees, with solitary bee richness and abundance associated with surrounding bare soil and woody structures [144]. Temporal complementarity between habitats was also reported, with within-field cover crop strips supporting wild bees earlier in spring and field margins becoming increasingly important in May and June [145]. Responses to surrounding landscape elements also differed among habitat types. Flower plantings and field margins benefited from nearby extensively managed grasslands and woodland edges, whereas near-natural grasslands responded more strongly to ruderal habitats [146]. Local flower richness consistently promoted bee communities across habitat types, while landscape composition influenced how different habitats contributed to overall diversity [140,146].

Pollinator responses to landscape composition also differed among taxonomic groups. Butterflies and syrphids showed contrasting responses to vegetation structure, with butterfly richness and abundance associated with herbaceous cover and plant species richness, whereas syrphid richness was similarly promoted by herbaceous vegetation and floral richness, but their abundance was more strongly associated with woody vegetation and landscape connectivity [147], whereas butterfly abundance also increased in landscapes with higher forest cover [148]. Hoverflies showed additional responses to the spatial arrangement of habitats, with their richness and abundance differing between hedgerows and nearby wheat fields [149]. Differences among pollinator groups were also reported at broader landscape scales, where bumble bees, solitary bees and hoverflies showed contrasting responses to habitat quantity, quality and floral resources [150].

Forest edges and grasslands within a 1 km radius supported higher pollinator abundance and richness than wildflower strips and sunflower monocultures, while proximity to forests and greater forest cover also enhanced bumble bee richness and abundance [151,152]. The positive influence of woody and semi-natural habitats was also observed in tropical agricultural systems, where increasing forest cover and the proximity of natural shrublands promoted wild bee communities, whereas flower flies were more strongly associated with landscape diversity and edge density [153,154]. The importance of forest habitats extended beyond foraging resources. Cavity-nesting insects have highlighted the importance of woody habitats as nesting and foraging resources within agricultural landscapes [155]. Responses to agricultural intensification further emphasised the importance of semi-natural habitats. Wild bee richness, abundance and functional diversity declined with increasing agricultural land cover, whereas semi-natural elements such as extensive grasslands, hedgerows and fallows, together with greater flower cover, promoted richer and more abundant wild bee communities. Species belonging to the family Colletidae were particularly sensitive to agricultural intensification and showed a strong association with semi-natural habitats [156]. Landscape simplification also modified bee community structure by increasing the dominance of a few generalist species, indicating that pollinator abundance alone may not adequately reflect changes in community diversity [157], while greater cover of mass-flowering oilseed rape was associated with lower bee richness and abundance within crop fields, with weaker effects of surrounding semi-natural habitat and no comparable response among non-bee pollinators [158]. Changes in landscape composition were reflected not only in pollinator communities but also in crop pollination. Greater forest cover within a 500 m radius increased wild pollinator abundance and functional richness in blueberry farms, with associated improvements in fruit size and yield, although responses differed between cultivars [159].

The effectiveness of local conservation measures also depended on the surrounding landscape. At the landscape scale, wild bee abundance and richness also increased with the area of local conservation interventions, while crop richness and crop evenness positively affected specific wild bee groups, whereas semi-natural habitat area and edge density showed no significant effects [160]. Landscape context also influenced the use of floral resources. A greater proportion of non-productive agri-environmental schemes, such as flower strips, was associated with higher plant species richness in bumble bee pollen loads, particularly in smaller fragmented grasslands [161]. Wildflower strips were more effective when floral resources were available in the surrounding landscape, although responses differed between solitary bees and bumble bees [162]. Their effectiveness also depended on their size and the surrounding landscape context. Smaller flower fields located in landscapes with a high proportion of semi-natural habitats promoted greater pollinator movement into adjacent oilseed rape fields, whereas larger flower fields were more effective in simplified landscapes with limited semi-natural habitat [163]. However, increasing the proportion of surrounding semi-natural habitats did not always enhance pollinator abundance within flower strips. A greater proportion of surrounding semi-natural habitats reduced insect abundance within flower strips, suggesting a dilution effect, whereas high-quality semi-natural habitats enhanced total insect and pollinator abundance [164]. Similarly, local habitat quality remained important. Perennial wildflower strips supported richer and more abundant wild bee communities when local habitat quality was high, particularly where flower-rich forb communities were maintained and grass cover remained low. Landscape heterogeneity further increased the occurrence of Red List species, highlighting the complementary roles of local habitat quality and surrounding landscape structure [165].

In Mediterranean olive groves, low-intensity ground cover management combined with greater landscape complexity promoted more diverse and less specialised plant–floral visitor interaction networks, highlighting the complementary importance of local floral resources and surrounding semi-natural habitats for maintaining resilient pollination networks [166]. Responses to landscape context also differed among pollinator groups. In strawberry fields, small wild bee abundance declined with increasing distance from forest or hedgerow margins towards the crop interior, whereas large bees and honey bees showed no comparable decline [167]. Landscape heterogeneity further promoted pollinator diversity. Agricultural landscapes characterised by smaller fields, longer field margins and structurally diverse crop mosaics supported higher bee and butterfly diversity than simplified monocultures [168]. The benefits of agri-environment schemes were also greater in structurally diverse landscapes. Bumble bee communities also responded to management, as species richness was higher in organically and environmentally friendly managed field margins than under conventional management, while flower cover was positively associated with all bumble bee groups [169]. Similarly, pollinator diversity increased with the proportion of semi-natural habitats and was consistently higher in landscapes maintaining small fields and extensive field margins than in consolidated agricultural landscapes [170]. In sunflower systems, both floral strips and semi-natural habitats supported higher taxonomic and functional diversity of wild bees than adjacent crop fields, with semi-natural habitats providing the greatest benefits [171]. These landscape effects influenced the performance of habitat enhancement measures. Wildflower strips depended on landscape context, promoting pollinator visitation and crop productivity in heterogeneous landscapes but showing no consistent benefits in simplified agricultural areas [172]. Likewise, flowering borders in strawberry fields enhanced bee visitation and fruit production primarily in landscapes with intermediate natural habitat cover, whereas their benefits were limited in highly simplified or highly natural landscapes [173].

Finally, orchard management practices also influenced the effectiveness of habitat enhancement measures. Landscape effects were not the only factors influencing the performance of habitat enhancement measures. In apple orchards, wildflower strips did not significantly affect bee abundance, richness or diversity, although hoverfly richness and diversity increased [174]. Overall, these findings indicate that pollinator responses should be evaluated at the landscape scale, as local increases in abundance may reflect the redistribution of individuals rather than true population growth [175].

## 4. Discussion

Overall, agroecological infrastructures generally support pollinator communities in agricultural landscapes. However, their effectiveness is highly context-dependent and varies according to habitat characteristics, landscape composition and the ecological requirements of different pollinator taxa. Consequently, no single habitat enhancement strategy consistently benefits all pollinator groups. Rather, maintaining a diversity of complementary habitats that provide floral resources, nesting sites and ecological connectivity is more likely to support diverse pollinator communities and the ecosystem services they provide [21,176].

The effectiveness of agroecological infrastructures depends largely on the landscape context in which they are established and on how they are managed. Their capacity to support pollinator communities is influenced not only by the presence of habitat elements, but also by factors such as habitat size, floristic composition, vegetation structure, management practices and the availability of surrounding semi-natural habitats [25,176]. Consequently, similar habitat enhancement measures may produce contrasting outcomes depending on local ecological conditions, highlighting the importance of adopting site-specific management strategies rather than relying on standardized conservation interventions.

These interacting factors help explain the considerable variability observed among studies and agricultural systems.

Among the factors influencing the success of agroecological infrastructures, landscape context appears to play a particularly important role. In structurally simple and intensively managed agricultural landscapes, habitat enhancement measures generally provide greater benefits by increasing the availability of floral resources and nesting opportunities. Conversely, in more heterogeneous landscapes already characterized by a high proportion of semi-natural habitats, the additional benefits of local interventions may be less pronounced, as pollinators can already exploit a wider range of resources across the landscape [176,177,178].

Differences in the ecology and behaviour of pollinator groups contribute to contrasting responses to agricultural habitats and management practices [15,179]. Different pollinator species may exploit complementary floral resources by visiting different flowers or inflorescences within the same plant, thereby improving the overall quality and quantity of pollination. In addition, non-bee pollinators often exhibit broader activity periods and can provide pollination services under weather conditions or at times of the day when bees are less active [11]. Pollinator responses to agricultural environments differ among taxa and crop systems because pollinator groups vary in their ecological traits, resource requirements, and life-history characteristics [15,179,180]. For example, bees generally show a stronger dependence on the availability of floral resources and nesting sites associated with surrounding semi-natural habitats, whereas non-bee pollinators appear less affected by increasing isolation from these habitats [11,15]. Despite their demonstrated contribution to crop pollination, the ecological role and management requirements of many non-bee pollinators remain poorly understood, highlighting an important research priority [15,179].

The geographical and crop-specific distribution of the selected studies (Figure 1) shows that Germany and the United States have produced the highest number of publications across the various crop categories included in this review. This pattern may partly reflect the implementation of agri-environmental policies and pollinator conservation programmes in these countries. In Germany, agri-environmental measures supported by the Common Agricultural Policy (CAP) have promoted the establishment of semi-natural habitats that benefit pollinators and biodiversity [21], whereas in the United States, the Conservation Reserve Program has encouraged the allocation of agricultural land to habitats providing floral and nesting resources for pollinators [181]. This concentration of research has substantially advanced current knowledge on agroecological infrastructures, but it also indicates that much of the available evidence has been generated under specific environmental, climatic and policy contexts.

Although studies conducted in the Mediterranean Basin accounted for 20.30% of the studies included in this review, this geographical region remains comparatively underrepresented considering its ecological importance. The Mediterranean Basin is recognized as one of the world’s biodiversity hotspots [27,28], characterized by exceptionally high levels of plant endemism and pollinator diversity.

The peculiar climatic conditions and the high floristic and faunistic biodiversity that characterize the Mediterranean basin suggest that this context represents a highly advantageous, yet structurally complex, environment for the experimentation of agroecological practices [182]. Unlike temperate zones, where environmental factors are more uniform, the Mediterranean Basin presents unique macro-climatic challenges, such as severe summer droughts and erratic rainfall patterns, which directly dictate the phenology of both crops and wild flora. This climatic distinctiveness means that the establishment, survival, and resource-provisioning window of agroecological infrastructures, such as flower strips or managed margins, are subjected to completely different ecological pressures [182].

Despite this, the Mediterranean Basin is still underexplored by the scientific literature regarding the use of agroecological infrastructure for the protection of pollinators [25].

Another important imbalance concerns the agricultural systems in which agroecological infrastructures have been implemented. Although many practices to support pollinators have proven effective, most studies have focused on herbaceous cropping systems (Figure 1). Conversely, arboreal cropping systems remain comparatively underrepresented, despite offering potentially favourable ecological conditions, including greater structural heterogeneity and longer growing seasons [183]. This imbalance in the literature limits the transferability of current knowledge to arboreal cropping systems. Furthermore, the interaction between cultivated trees, spontaneous vegetation and pollinating insects is inherently more complex than in annual cropping systems and remains comparatively less investigated.

The studies included in this review demonstrate that agroecological infrastructures can also provide important ecological benefits in arboreal cropping systems [32,55,61,62,68,69,70,91,92,93,112].

Landscape context plays a fundamental role in determining the effectiveness of agroecological infrastructures by influencing pollinator occurrence and the availability of nesting and floral resources [184]. Wooded areas and non-forest woody vegetation provide critical landscape features, offering nesting habitats, refuge during periods of floral scarcity, and non-floral materials used by around 32% of bee species for nest construction and protection [178,185]. Because many small-bodied bees have limited foraging ranges, the spatial proximity of woody habitats and other floral resources to crops is critical for maximizing the effectiveness of agroecological infrastructures [186].

This spatial constraint underlies the distance-dependent decline in the effectiveness of flower strips, where proximity directly influences their effectiveness on crop yields [177] and seed fertilisation [73]. Although flower strips provide significant support to pollinators in intensively managed agricultural landscapes [74], their effectiveness is strongly influenced by the surrounding landscape context and the availability of semi-natural habitats [176].

To maximize the benefits of agroecological infrastructures, conserving spontaneous vegetation along field margins represents an effective strategy for supporting pollinator diversity and complementing the ecological functions of sown flower strips [187]. However, the long-term effectiveness of these habitats depends on appropriate management. Rotational mowing regimes that leave portions of the vegetation temporarily unmown help maintain habitat heterogeneity and continuous floral resources throughout the season, thereby supporting a wider range of pollinator taxa [188,189,190,191].

The selection of plant species is equally important. Seed mixtures should be designed according to the ecological requirements of local pollinator communities, taking into account floral preferences, flowering phenology and the temporal continuity of floral resources, rather than relying solely on overall attractiveness [192,193,194,195,196,197]. Incorporating key resource plant species can support a broad range of pollinator taxa, including species of conservation concern [122], while the inclusion of rare arable plants may further diversify available floral resources and create additional ecological niches for wild bees [198].

Ensuring a continuous supply of floral resources throughout the growing season is equally important for sustaining pollinator communities. In Mediterranean agroecosystems, where summer floral resources are often limited, the inclusion of plant species with extended flowering periods can help bridge critical phenological gaps [199]. In addition to sown species, spontaneous plants and agricultural weeds can provide valuable nectar and pollen resources for a wide range of pollinators and should therefore be considered as complementary components of habitat management rather than systematically removed [200,201].

Beyond their role in supporting pollinators, agroecological infrastructures provide multiple ecosystem services. Semi-natural habitats contribute to biodiversity conservation, improve soil and water quality, and enhance the aesthetic value of agricultural landscapes [202]. Likewise, agri-environment schemes established for other conservation objectives may also provide substantial benefits for pollinators. For example, bird-targeted agri-environment schemes in Belgium were shown to supply abundant floral resources for flower-visiting insects [203]. Furthermore, increasing floral diversity within field margins can simultaneously enhance pollination and conservation biological control, contributing to reduced crop damage and improved crop productivity [204,205].

## 5. Conclusions

This review highlights the important role of agroecological infrastructures in supporting pollinator communities within agricultural landscapes. However, their effectiveness is highly context-dependent and influenced by landscape composition, habitat quality, specific management practices, ecological maturation over time and the ecological requirements of different pollinator groups. Crucially, an increase in pollinator abundance or richness within agroecological infrastructures does not automatically translate into enhanced crop pollination or increased yield within adjacent field interiors, as pollinator spillover depends heavily on species-specific foraging behaviour, spatial constraints, and crop identity.

Rather than relying on isolated or standardized interventions, integrating complementary habitat elements within agricultural landscapes appears to provide the greatest potential for supporting pollinator diversity and the ecosystem services they deliver. Sown flower strips offer rapid, high-density floral resources for generalist species, whereas permanent hedgerows, semi-natural habitats, and unmanaged field margins provide long-term stability, essential overwintering sites, and specialized resources for rare or oligolectic taxa. Furthermore, when selecting plant species, priority should be given to phenological complementarity, local adaptation and diversity of functional traits rather than simply species richness. The adoption of practical strategies, such as Marketable Habitat Enhancement Plants (MHEP) or rotational or meandering mowing regimes, can successfully address resource constraints whilst ensuring economic and operational viability for farmers.

At the same time, this review identifies three critical knowledge gaps that warrant future empirical investigation. First, a pronounced geographical and agricultural bias persists, as the current literature remains heavily focused on annual cropping systems, predominantly in Western Europe and North America; future research must therefore focus on Mediterranean agroecosystems, a global biodiversity hotspot exposed to severe climatic stressors, and on perennial tree crops, which remain under-represented despite their structural potential for long-term integration into the habitat.

Second, expanding the taxonomic breadth beyond bees is essential, as non-bee pollinators such as hoverflies, butterflies, and beetles require more in-depth study regarding their specific foraging, larval development, and nesting requirements in managed landscapes.

Finally, there is considerable uncertainty regarding the temporal and economic dynamics, as long-term studies evaluating habitat maturation beyond 3–5 years and comprehensive economic assessments evaluating the net cost–benefit balance of ecosystem services versus land take remain scarce.

Future research should expand beyond herbaceous cropping systems and the geographical regions currently dominating the literature, with greater attention to the Mediterranean Basin and arboreal agroecosystems, as well as to non-bee pollinators, which remain comparatively understudied. A more integrated understanding of how landscape context, habitat management and pollinator ecology interact will be essential for designing effective conservation strategies capable of reconciling agricultural production with biodiversity conservation.

## Figures and Tables

**Figure 1:**
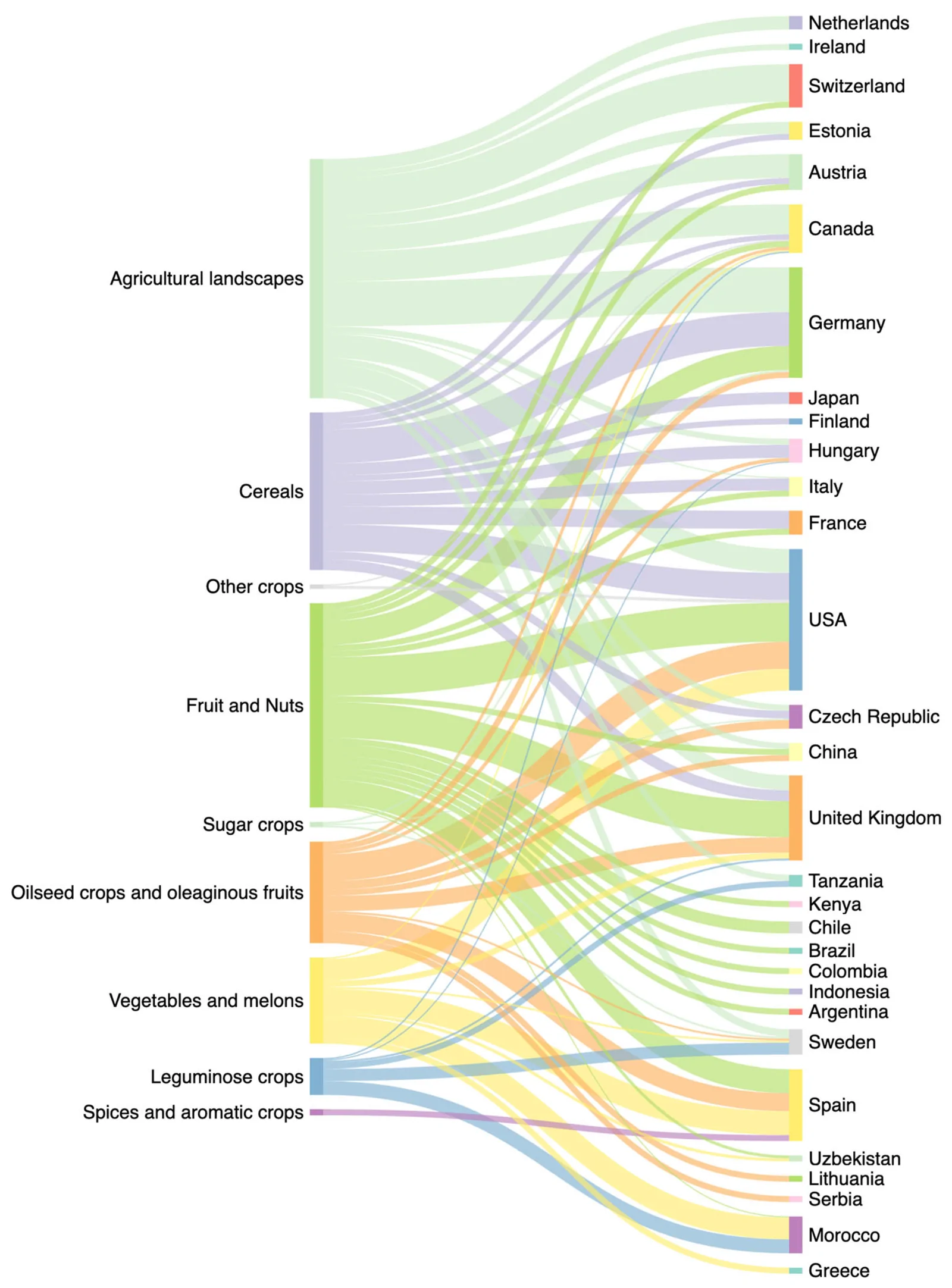
Distribution of the reviewed studies across crop categories and geographical areas. Crop categories were defined at the Group level according to the FAO Indicative Crop Classification Version 1.2 (ICC 1.2) [31]. For studies including crops belonging to more than one crop category, each study was proportionally allocated among the relevant categories so that its total contribution equalled one. “Other crops” includes cotton, flax and forage. “Agricultural landscapes” includes studies conducted in agricultural environments for which no specific crop was reported, as well as studies conducted in pastures, which were not assigned to a specific ICC 1.2 crop group.

**Figure 2:**
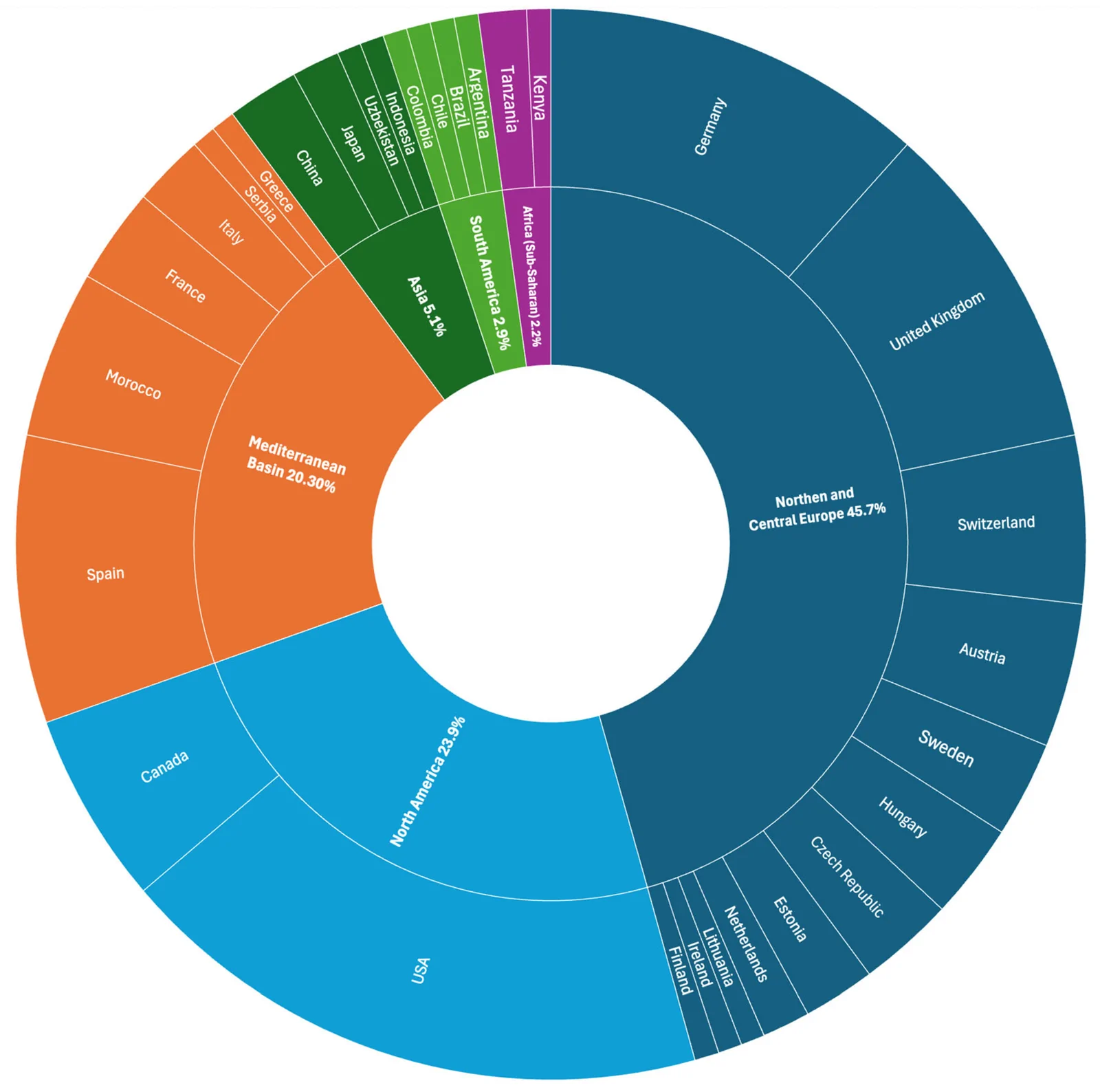
Hierarchical distribution of the scientific literature analysed according to geographical location. The inner circle indicates the macro-geographical regions, while the outer circle shows the individual countries.

**Table 1:** Summary of the main categories identified in the reviewed literature, including representative agroecological infrastructures and the most frequently investigated response variables associated with each category.

Main Category	Representative Agroecological Infrastructures	No. of Studies	Response Variables
Floral resource enhancement	Flower and wildflower strips, wildflower fields and areas, wildflower mixtures, flowering borders, pollination reservoirs, planted flower plots, flowering refugia and floral enhancement plots.	55	Pollinator abundance, richness and diversity, visitation rates, community composition and pollination services.
Semi-natural herbaceous habitat enhancement	Field margins, grasslands, meadows, grassy and herbaceous strips, prairie strips and other herbaceous semi-natural elements.	30	Pollinator abundance, richness and diversity, community composition, habitat use, spillover and pollination services.
Woody habitat enhancement	Hedgerows, woodland and forest edges.	10	Pollinator abundance, richness and diversity, community composition, habitat use, floral and nesting resources.
Habitat enhancement schemes	Agri-environment schemes (AES) and Farming with Alternative Pollinators (FAP)	13	Pollinator abundance, richness and diversity, visitation rates, pollination services and crop yield.
Within-field vegetation enhancement	Cover crops, artificial field defects, inter-row vegetation, herb cover and mid-field strip	8	Floral resource availability, pollinator abundance, richness and diversity, visitation rates and habitat use.
Multiple strategies	Combinations of flower strips, field margins, grasslands, meadows and pastures, hedgerows, woodland and forest edges, shrublands, residual habitats, drainage ditches and stone walls	28	Pollinator abundance, richness and diversity, community composition, habitat use, floral resources, visitation rates and pollination services.

## Data Availability

The raw data supporting the conclusions of this article will be made available by the authors on request.

## Supplementary Materials

The following supporting information can be downloaded at: https://www.mdpi.com/article/10.3390/insects17100987/s1, Figure S1. The systematic screening of the relevant literature was conducted in accordance with PRISMA guidelines using studies retrieved from major scientific databases; Table S1. Overview of published studies assessing the effects of agroecological infrastructures on pollinator communities, sampling methods, and crop systems across different geographical regions; Table S2. Distribution of pollinator response directions to agroecological infrastructures across the studies included in the review, according to pollinator group; Table S3. Distribution of pollinator response directions to agroecological infrastructures across the studies included in the review, according to the main agroecological infrastructure category.
