# Supplementary material for: Pollinator Conservation Strategies: The Effectiveness of Agroecological Infrastructure in Agricultural Environments
Source: Insects. 2026 Sep 24;17(10):987. doi: 10.3390/insects17100987 (PMC13705111; doi:10.3390/insects17100987)
Supplement: Supplementary file 1 [file insects-17-00987-s001.zip › insects-4520208-supplementary.pdf]

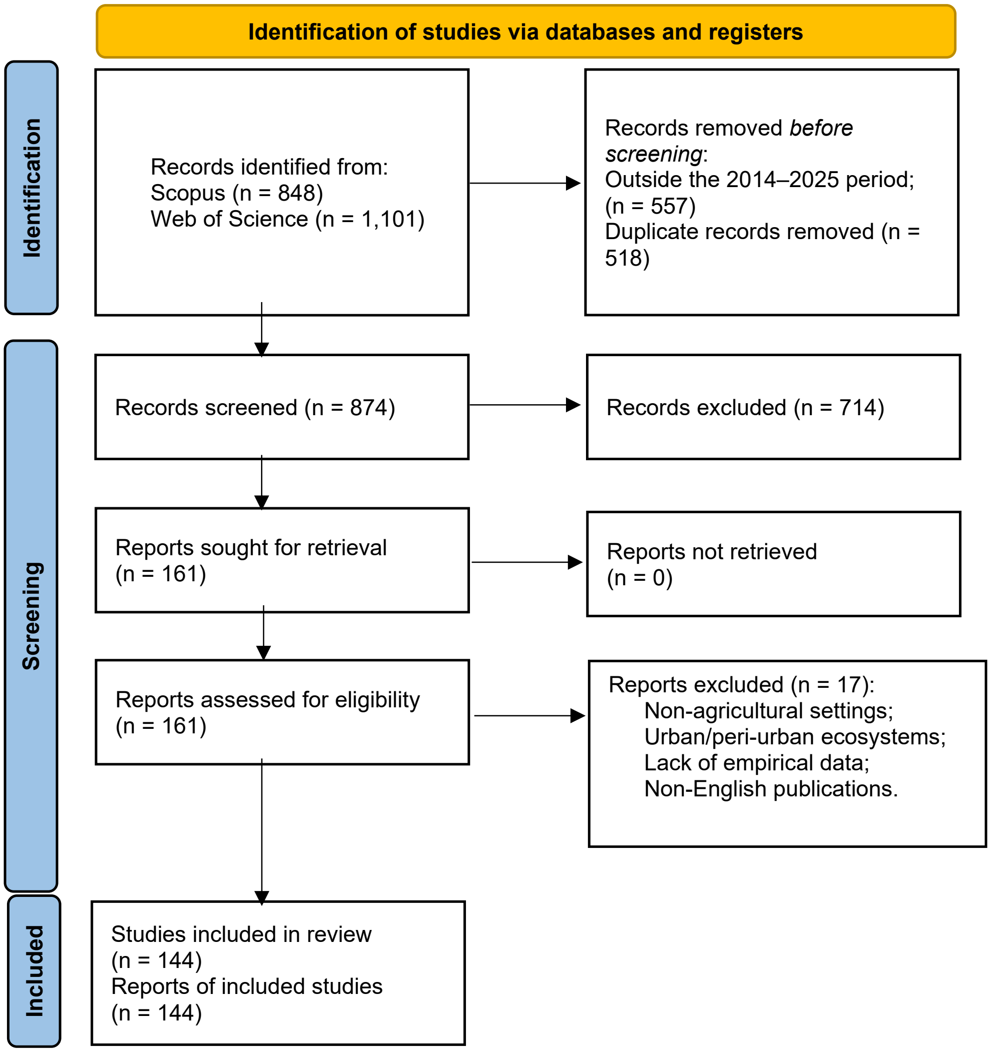

Figure S1. The systematic screening of the relevant literature was conducted in accordance with PRISMA guidelines using studies retrieved from major scientific databases.

Table S1. Overview of published studies assessing the effects of agroecological infrastructures on pollinator communities, sampling methods, and crop systems across different geographical regions.

Type of strategies	*Crop*	*Pollinators group*	*Effect on beneficials*	*State/ Region*	*References*
Fields margins	*Wheat, alfalfa, maize*	Butterflies and Syrphidae	Butterflies responded to herbaceous vegetation, whereas syrphids also responded to woody vegetation and hedge connectivity.	Italy	[147]
	Coriander	Wild bees, Syrphidae and Coleoptera	Enhanced pollinator visitation and improved coriander seed production and seed quality.	Spain	[123]
	Oilseed rape	*A. mellifera, Bombus* spp., wild bees and Syrphidae	Pollinator visitation declined with increasing distance from field margins, except for honey bees.	United Kingdom	[59]
	Maize and soybean	*Bombus* sp., wild bees and Diptera	Agricultural intensification altered wild bee community composition, favouring dominant generalists.	USA	[157]
	Agricultural landscapes	*Bombus* spp.	Bumble bee abundance and diversity were similar between field margins and semi-natural habitats.	Canada	[23]
	Strawberry	Wild bees and *A. mellifera*	Small wild bee abundance declined with distance from field edges, whereas large bees and honey bees did not.	Canada	[167]
	Spinach	*A. mellifera,* wild Apidae, Megachilidae and mining bees	Restored field margins increased wild bee abundance and bee richness and diversity, while bee community composition differed between herbaceous and shrubby margins.	Spain	[116]
	Oilseed rape	Hymenoptera, Lepidoptera and Diptera	Pollinator richness and evenness were higher in traditional than consolidated farmland, while abundance did not differ.	China	[170]
	Agricultural landscapes	*A. mellifera*, wild bees and Syrphidae	Pollinator richness declined with land-use intensification but increased with local flower density.	Tanzania	[134]
	Agricultural landscapes	Wild bees, Lepidoptera and wasps	Structurally diverse crop mosaics and field margins supported higher pollinator diversity than monocultures.	China	[168]
	Winter wheat, barley, sugar beet and oilseed rape	Bees, wasps, Lepidoptera and Syrphidae	Increased the abundance of bees, wasps and butterflies near field margins.	Czech Republic	[60]
	Cereal	*Bombus* spp.	Bumble bee richness and abundance were higher in margins adjacent to forest and increased with forest cover.	Estonia	[152]
	Buckwheat	*A. mellifera, Bombus* spp., wild bees, wasps, Syrphidae, other fly, butterflies and beetles	Increased flower-visiting insects and enhanced buckwheat seed set.	Japan	[58]
	Melon	*A. mellifera,* wild bees, Syrphidae, and other pollinators	Increased pollinator abundance and richness without improving melon yield.	Spain	[54]
	Melon	*A. mellifera,* Apidae and wild bees	Managed herbaceous margins supported higher bee abundance in the first year, whereas managed shrubby margins became more effective in the second year after establishment.	Spain	[82]
	Bean	*A. mellifera*, Syrphidae, wild bees and Lepidoptera	Insect pollination significantly increased bean yield through greater pod production and seed set.	Tanzania	[57]
	Buckwheat	*A. mellifera, A. cerana japonica, Bombus* spp., wild bees, wasps, Syrphidae, other flies, Lepidoptera and Coleoptera	Insect abundance increased with co-flowering plant richness in autumn, but not in summer.	Japan	[99]
	Wheat, barley, maize, sunflower and oilseed rape	*A. mellifera, Bombus* spp., mining, trap-nesting bees, Syrphidae and Lepidoptera	Pollinator abundance increased with flower abundance, whereas responses to margin age differed among taxa.	Hungary	[80]
	Tomato, basil and watermelon	Wild bees	Increased nest density and species richness of soil-nesting bees.	California	[90]
	Tomato	*A. mellifera*, wild bees, Diptera, Lepidoptera and *Bombus* sp.	Enhanced flower visitor abundance compared with naturally vegetated margins, while wild bee visitation to tomato flowers remained unchanged.	Greece	[109]
	Potato	Wild bees, *Bombus* spp., *A. mellifera,* Syrphidae and other Diptera	Increased pollinator abundance, with limited spillover into adjacent potato crops.	USA	[53]
Grass field margins, pollen and nectar margins and wildflower patches	*Wheat, oilseed rape and field beans*	*A. mellifera, Bombus* spp. and ground beetles	Crop yield increased from the fourth year onwards with higher pollinator abundance.	United Kingdom	[94]
Fields margins and flower strips	*Maize*	Lepidoptera	Flower strips supported richer butterfly communities, with responses primarily driven by flowering plant richness and landscape context.	Germany	[102]
	Agricultural landscapes	*Bombus* spp. and solitary bees	Bumble bees responded rapidly to habitat creation, whereas solitary bees showed slower population responses requiring landscape-scale evaluation.	Netherlands	[175]
	Sugar beet, onion and oilseed rape	Solitary bees and wasps	Solitary bee nesting tended to increase near flower strips, whereas wasps showed no response.	Sweden	[136]
Fields margins, meadows and semi- natural habitats	*Agricultural landscapes*	Wild bees and Syrphidae	Field margins supported the highest bee abundance and species richness owing to greater floral resource availability.	Canada	[42]
Field margins, grassland, herbaceous and grassy and strips	*Agricultural landscapes*	*A. mellifera, Bombus* spp. and wild bees	Flower visits increased with plant species richness across all bee groups, which showed complementary preferences for distinct key plant species.	Switzerland	[122]

Field margins, semi-natural grasslands and forest edges	*Cereal*	Lepidoptera	Butterfly richness and abundance increased with greater forest cover, with responses varying among habitats.	Finland	[148]
Wildflower strips	*Strawberry*	*Bombus* spp., wild bees, Syrphidae and *A. mellifera*	Increased pollinator visitation to adjacent strawberry crops.	Scotland	[56]
	Strawberry	*A. mellifera* and wild bees	Bee visitation and crop yield increased only in landscapes with intermediate natural habitat cover.	USA	[173]
	Strawberry	*A. mellifera*, *Bombus* spp., wild bees and Syrphidae	Enhanced pollination services and wild bee visitation near flower strips.	Switzerland	[73]
	Agricultural landscapes	*A. mellifera*, wild bees and Syrphidae	Pollinator abundance declined with strip age due to reduced floral resources, although older strips may still provide nesting and overwintering habitat.	Switzerland	[79]
	Courgette	Solitary bees, *Bombus* spp. and *A. mellifera*	Wildflower richness supported crop pollinators without reducing crop visitation.	United Kingdom	[71]
	Agricultural landscapes	Wild bees	Certain plant species supported high wild bee richness, while additional species supported other, including rarer bee species.	USA	[119]
	Agricultural landscapes	Wild bees	Enhanced reproductive success, particularly in small solitary bees with limited foraging ranges.	Switzerland	[89]
	Agricultural landscapes	*Bombus* spp., Syrphidae and butterflies	Bumblebees and hoverflies preferred different flower mixtures, while butterflies responded to flower richness.	Netherlands	[114]
	Sunflower	*A. mellifera* and wild bees	Wild bee visitation increased in the second year, while seed set and estimated yield were higher in fields with flower strips.	Spain	[77]
	Winter wheat, sugar beet and maize	Wild bees and Lepidoptera	Wild bee and butterfly richness and abundance were higher in flower strips than in reference field margins.	Germany	[86]
	Agricultural landscapes	*A. mellifera* and *Bombus* spp.	Greater floral diversity altered pollinator interaction networks, reducing connectance and niche overlap among bee species.	United Kingdom	[33]
	Agricultural landscapes	Wild bees	Wild bee richness depended on local floral resource quality, whereas Red List species were mainly influenced by surrounding landscape heterogeneity.	Germany	[165]
	Alfalfa, barley, maize, sunflower and wheat	*A. mellifera*, wild bees and Syrphidae	Bee abundance was highest, while hoverfly abundance exceeded that in crop fields.	Hungary	[141]
	Winter squash and sunflower	*Bombus* spp., *A. mellifera* and wild bees	Proximity to wildflower strips did not enhance pollination services.	USA	[72]
	Oilseed rape	Wild bees	Increased pollinator diversity and evenness, whereas semi-natural grasslands support higher abundance.	Lithuania	[67]
	Strawberry	*Bombus* spp., wild bees, *A. mellifera* and Syrphidae	Companion flowering plants increased overall flower visitation but diverted pollinators from strawberry flowers, without affecting marketable fruit yield.	United Kingdom	[108]
	Cherry	*A. mellifera*, *Bombus* spp., solitary bees and Syrphidae	Enhanced pollinator abundance, richness and diversity after anthesis, especially in the third year.	United Kingdom	[92]
Wildflower areas	*Agricultural landscapes*	Wild bees and Syrphidae	Connectivity increased pollinator richness in simple landscapes but reduced it in complex landscapes.	Germany	[137]
Wildflower mixtures	*Agricultural landscapes*	Wild bees and Syrphidae	A small number of key plant species supported most flower visitors, and seed mixtures differed in their attractiveness to wild bees and hoverflies.	Germany	[118]
Wildflower strips and semi natural vegetation	*Sunflower*	*A. mellifera* and wild bees	Pollinator visitation and sunflower productivity increased in the second year in the more heterogeneous landscape.	Spain	[172]
Wildflower fields and strips	*Agricultural landscapes*	Wild bees, Syrphidae and butterflies	Increased pollinator abundance and species richness, particularly in flower-poor landscapes.	Hungary	[97]
Flower Strips	*Almond*	*Osmia lignaria*, wild bees and *A. mellifera*	Enhance the use of inter-row flower strips by *Osmia lignaria* and diverse wild bees, particularly within 40 m of nesting sites	California	[61]
	Agricultural landscapes	*Bombus* spp., solitary bees and Syrphidae	Increased bumble bee abundance across the surrounding landscape, while hoverflies and solitary bees responded according to landscape context and flower strip quality.	Sweden	[115]
	Agricultural landscapes	*A. mellifera*, *Bombus* spp. and wild bees	Bee abundance and richness generally increased, with bumble bee responses varying among countries.	Germany, Sweden, Netherlands and the United Kingdom	[162]
	Agricultural landscapes	Wild bees, wasp hoverflies and butterflies	Mixed flower strips supported greater hoverfly and butterfly diversity than *Phacelia* monocultures.	Czech Republic	[105]
	Apple	*A. mellifera*, *Bombus* spp., wild bees, Syrphidae, Diptera and Coleoptera	Increased wild insect visitation without improving fruit set.	United Kingdom	[91]
	Apple	*A. mellifera*, *Bombus* spp., wild bees, Syrphidae, Diptera and Coleoptera	Enhanced pollinator richness and supported both pollinators and natural enemies through multifunctional flower strips.	United Kingdom	[93]
	Cucumber	*Bombus impatiens, A. mellifera*, Syrphidae and wild bees	Higher pollinator abundance did not increase cucumber visitation, while yield increased only with *L. maritima.*	USA	[113]
	Oilseed rape	*A. mellifera*, *Bombus* spp., wild bees, Syrphidae and other flies	Pollinator spillover into oilseed rape depended on flower strip age, size and surrounding semi-natural habitat.	Germany	[163]
	Maize and winter cereals	Lepidoptera, *Bombus* spp. and wild bees	Long-term flower strip networks enhanced bee and butterfly richness, with specialist bees responding after >2 years.	Germany	[74]
	Melon	*A. mellifera*, wild bees, Syrphidae, Coleoptera and *Bombus* spp.	Bee visitation declined with increasing distance from the flower strip, with no effect on crop productivity.	Spain	[76]
	Apple	*Bombus* spp., *A. mellifera,* wild bees and Syrphidae	Hoverfly richness and diversity increased, with no significant changes in bee abundance, richness or diversity.	United Kingdom	[174]
	Winter wheat	Syrphidae, solitary bee and *Bombus* spp.	Organic wheat fields and flower strips supported higher pollinator abundance and richness than conventional fields.	Germany	[37]
	Peanuts and cotton	*A. mellifera, Bombus* spp. and wild bees	Wild bees foraged on both wildflower strips and adjacent crops, indicating complementary resource use.	USA	[130]

	Faba beans	*A. mellifera, Bombus* spp. and Lepidoptera	Flower strips redistributed pollinators within faba bean fields.	Sweden	[110]
	Faba beans	*A. mellifera* and *Bombus* spp.,	Enhanced bumble bee reproductive success and queen abundance across seasons.	Sweden	[38]
	Strawberries and winter squash	Wild bees	Wild bee richness increased with bloom density, while managed A. mellifera hives were associated with lower wild bee abundance and richness, with no effect on crop yield.	USA	[39]
	Winter wheat, spring barley, oilseed rape and maize	*A. mellifera, Bombus* spp. and solitary bees	Flower strips mainly supported honey bees, whereas semi-natural habitats sustained more diverse communities of solitary bees and bumble bees.	Czech Republic	[66]
	Agricultural landscapes	*Apis* spp., *Bombus* spp., wild bees and Syrphidae	Pollinator abundance increased with floral cover, while responses were strongly influenced by the quality of surrounding habitats.	Switzerland	[164]
	Cereals	Wild bees	Wild bee responses to flower strips depended on landscape context and bee sociality.	Germany	[144]
	Agricultural landscapes	Heteroptera, Lepidoptera and Syrphidae	Enhanced syrphid abundance and richness, increased Heteroptera richness, and promoted spillover into adjacent intensive grasslands	Austria	[51]
	Agricultural landscapes	*A. mellifera, Bombus* spp., Syrphidae, larger solitary bees and Lepidoptera	Species-rich mixtures extended flowering duration, whereas monocultures and low-diversity mixtures attracted the highest pollinator visitation.	Estonia	[106]
	Avocado	*A. mellifera*, wild bees, Syrphidae, wasps, other Diptera, Lepidoptera, Calliphoridae and coccinellids	Flower visitors were concentrated within the strips without increasing avocado fruit set.	Kenya	[112]
	Oat, pea, canola, soybean, wheat, maize, flax and forage	*Bombus* spp., *A. mellifera,* Coleoptera, wild bees and Syrphidae	Enhanced bee abundance, whereas syrphids were more abundant in control field margins.	Canada	[75]
	Kiwi	*Osmia bicornis, A. mellifera, Bombus* spp., wild bees, Syrphidae and other pollinators	Higher floral cover reduced pollinator richness on kiwifruit flowers and altered their visitation patterns.	Italy	[111]
	Maize	*Bombus* spp., *A. mellifera* and other bees	Annual flower strips increased plant diversity and pollinator abundance, with delayed sowing maintaining these biodiversity benefits.	USA	[81, 104]
	Agricultural landscapes	*A. mellifera*, *Bombus* spp., wild bees, Syrphidae and Coleoptera	Maintained diverse floral resources while promoting bee spillover into adjacent fields.	Canada	[75]
	Avocado	*A. mellifera*, wasp, wild bees, Diptera, Coleoptera and Lepidoptera	Native flower strips supported diverse pollinator communities, increased visitation by non-bee pollinators to avocado flowers and promoted fruit production.	Chile	[32, 55]
Flower strips and Semi-natural grasslands	*Vineyard*	*A. mellifera, Bombus* spp., wild bees, Diptera, Lepidoptera and Coleoptera,	Semi-natural grasslands supported higher wild bee diversity, while flower strips enhanced the abundance of generalist flower-visiting insects.	Austria	[65]
	Sunflowers	*A. mellifera, Bombus* spp. and wild bees	Wild bee species and functional richness were higher than in sunflower fields, with semi-natural habitats supporting greater functional diversity.	Spain	[171]
Flower strip and hedgerows	*Apple*	*A. mellifera,* wild bees and Syrphidae	Flower strips enhanced wild bee diversity, whereas hedgerows complemented floral resources throughout the season.	Germany	[62, 68, 69]
	Apple	*A. mellifera, Bombus* spp., wild bees and Syrphidae	Promoted more constant plant–pollinator interactions throughout the season without reducing apple pollination.	Germany	[70]
Flowering border crops	*Agricultural landscapes*	Bees, Syrphidae, Bombyliidae, and Lepidoptera	Native perennial plantings supported the highest overall pollinator abundance and richness.	USA	[121]
Hedgerows	*Agricultural landscapes*	Bees and Syrphidae	Restored native hedgerows enhanced pollinator occurrence, particularly of specialized species.	California	[88]
	Agricultural landscapes	Bees and Syrphidae	Enhanced pollinator persistence, colonization and species richness over time	California	[87]
	Wheat, barley, oilseed rape and field beans	*Bombus* spp.	Roadside hedgerow margins supported higher bumble bee abundance.	United Kingdom	[139]
	Sunflower	Wild bees	Supported sunflower-specialist wild bees but did not consistently enhance crop pollination.	California	[135]
	Agricultural landscapes	Wild bees	Provided floral resources but did not improve nesting habitat for ground-nesting bees.	California	[143]
	Avocado	Wild bees, *A. mellifera,* Syrphidae, wasps and Coleoptera	Increased bee species diversity and slightly increased bee abundance; provided floral resources for diverse flower-visiting insects.	USA	[41]
	Wheat	Syrphidae	Hoverfly richness and abundance were higher in the adjacent wheat field, while flower-rich margins provided important foraging resources.	Hungary	[149]
	Agricultural landscapes	Wild bees and *Bombus* spp.	Hedgerow proximity enhanced wild bee colonisation, while persistence depended on local floral diversity and species traits.	California	[138]
	Winter wheat	*Bombus* spp., *A. mellifera*, Syrphidae and solitary bees	High-quality hedgerows supported more bumble bees, while flower-rich understories enhanced hoverfly abundance.	United Kingdom	[40]
Hedgerow and field margins	*Agricultural landscapes*	*A. mellifera* and wild bees	Grass margins supported higher wild bee diversity than hedgerows and provided important herbaceous floral resources.	Canada	[44]
Hedgerows, drainage ditches, stone walls and grassy margins	*Agricultural landscapes*	*Bombus* spp., *A. mellifera*, solitary bees and Syrphidae	Social bees were associated with hedgerows, solitary bees with floral richness, and hoverflies with drainage ditches.	Ireland	[150]
Hedgerows and flowering weeds	*Wheat, barley, oat, rye or triticale*	Solitary bees, *Bombus* spp., Syrphidae, Lepidoptera, Coleoptera, Diptera and other Hymenoptera	Wild plants in hedgerows and weeds enhanced most flower-visiting insects, whereas legume intercrops had limited additional effects.	France	[103]

Farming with Alternative Pollinators (FAP)	*Melon*	*A. mellifera,* wild bees and wasps	Flower-visitor abundance and species richness were higher in FAP than in control fields, with 16.5% of wild bees and wasps showing spillover from field margins to melon.	Morocco	[126]
	Faba bean	*A. mellifera,* wild bees, wasps, Syrphidae, Lepidoptera and Coleoptera	FAP increased wild pollinator abundance and species richness and supported diverse flower visitors.	Morocco	[128]
	Faba bean and eggplant	*A. mellifera, Bombus terrestris*, wild bees, wasps, Syrphidae and Lepidoptera	FAP fields supported higher wild pollinator richness than control fields and surrounding wild vegetation.	Morocco	[131]
	Faba bean and pumpkin	Wild bees	FAP approach did not significantly increase pollinator dietary breadth or the number of pollen genera collected.	Morocco	[129]
	Pumpkin, zucchini, faba bean, tomato, eggplant and apple	Wild bees, wasps, Lepidoptera, Syrphidae and other flies	FAP fields increased wild pollinator abundance and richness at the whole-field level.	Morocco	[132]
	Melon	*A. mellifera, Bombus terrestris*, wild bees, Syrphidae and wasp	Flower visitors and natural enemies were more abundant in FAP fields, while pest abundance was reduced and melon yield was significantly higher than in control fields.	Morocco	[127]
	Faba bean, melon, zucchini, pumpkin, eggplant, tomato and ~~e~~okra	*A. mellifera, Bombus terrestris,* wild bees, wasps, Lepidoptera, Syrphidae and other Diptera	MHEP supported higher abundance and diversity of flower visitors and natural enemies over a prolonged flowering period.	Morocco	[133]
	Cucumber and sour cherry	*A. mellifera* and wild bees	FAP increased pollinator diversity, doubled sour cherry yield and substantially increased cucumber income.	Uzbekistan	[125]
Agri-environment schemes (AES)	*Agricultural landscapes*	*Bombus* spp.	Increased bumble bee species richness through agri-environmental management.	Estonia	[169]
	Agricultural landscapes	Wild bees and *Bombus* spp.	Increasing the area of conservation interventions enhanced the richness and abundance of solitary and rare bees.	Germany	[160]
	Agricultural landscapes	*Bombus pascuorum* and *Bombus lapidarius*	Greater coverage was associated with higher pollen plant species richness in both bumblebee species.	Germany	[161]
	Winter wheat	Wild bees, *Bombus* spp., Syrphidae and wasps	Wild bee overwintering benefited from perennial flowering fields with low soil disturbance, whereas responses differed among pollinator groups.	Germany	[85]
	Agricultural landscapes	Solitary bees and wasps	Wasps benefit from AES, but solitary bees do not show immediate benefits	Germany	[155]
Low-input meadows and pastures, herbaceous and flower strips	*Agricultural landscapes*	Wild bees	Wild bee richness and abundance increased with flowering plant richness and differed among habitat types.	Switzerland	[142]
Woodland and rosaceous hedges and grass and flower strips	*Vineyard*	Coleoptera, Diptera, *A. mellifera,* wild bees and *Bombus* spp.	Different ecological infrastructures supported complementary pollinator communities.	Spain	[52]
Herbaceous and woody semi-natural habitats	*Agricultural landscapes*	*A. mellifera*, wild bees and Syrphidae	Bee abundance was highest in herbaceous semi-natural habitats and was strongly associated with herbaceous cover and key flowering plant groups.	Italy, Switzerland, Germany, United Kingdom	[101]
Broad-leaved and coniferous woodland, restored, species-rich and other grasslands	*Oilseed rape*	*A. mellifera*, wild bees, Syrphidae and *Bombus* spp.	Increasing mass-flowering crop cover reduced bee richness and abundance, while non-bee pollinators were less affected.	United Kingdom	[158]
Forest edges, hedgerows and permanent grasslands	*Winter cereals*	Wild bees	Wild bee communities differed in composition, with wooded habitats supporting unique bee–pollen interactions.	France	[64]
Conventionally and extensively managed meadows, flower strips, hedgerows and forest edges	*Agricultural landscapes*	Wild bees	Wild bee abundance and richness were highest in extensively managed meadows, with flower strips becoming more important later in the season.	Switzerland	[140]
Natural shrubland, plantation forest and flowering ground-cover	*Apple*	Wild bees	Wild bee abundance declined with increasing distance from natural shrubland, while plantation forest cover negatively affected abundance and species richness.	China	[154]
Forest fragments, pasture and abandoned crops or pastures	*Coffee*	*A. mellifera*, wasp, wild bees and Syrphidae	Forest cover increased bee richness and wasp richness and abundance, while landscape diversity and edge density influenced flower fly species turnover.	Brazil	[153]
Pollination reservoirs	*Blueberry*	Wild bees, *A. mellifera, Bombus* spp. and Syrphidae	Social and solitary bees differed in their use of plantings, while wild bee visitation to blueberry increased after four years.	USA	[120]
Restored prairie, deciduous forest patches, road and field edge	*Maize, soybeans, squash, pumpkins*	Wild bees	Restored native prairie supported higher wild bee abundance and species richness than crop fields, forest patches and edge habitats.	Canada	[46]

Near-natural grasslands, flower plantings and residual habitats	*Agricultural landscapes*	Wild bees and *Bombus* spp.	Wild bees and bumble bees increased in richness and abundance with local floral resources, while responses to landscape composition varied among habitats.	Germany	[146]
Weeds, other crops and hedgerows	*Plum, pear, apple and peach*	*A. mellifera, Bombus* spp., wild bees, wasps, Syrphidae and Lepidoptera	Flowering plant richness and blooming cover increased flower visitor abundance and shaped plant–pollinator network structure.	Colombia	[100]
Woodland, cropland and hedgerows	*Pasture fields*	*A. mellifera, Bombus* spp., mining bees, Syrphidae and Lepidoptera	Enhanced the abundance of mining bees, hoverflies and honeybees in intensive grassland landscapes.	United Kingdom	[63]
Grassland	*Agricultural landscapes*	Solitary bees, Syrphidae, *Bombus* spp. and Lepidoptera	Species-rich new grasslands enhanced abundance and richness of wild bees, bumble bees and syrphids, while permanent old grasslands remained the most suitable habitat for butterflies.	Austria	[83]
	Maize and soybean	Lepidoptera	Conservation grasslands supported higher grassland butterfly abundance than marginal grasslands.	USA	[49]
	Agricultural landscapes	Syrphidae, *Bombus* spp. and solitary bees	Grassland restoration rapidly enhanced pollinator abundance and species richness.	Austria	[45]
	Cereal and/or maize	Solitary bees, Syrphidae, *Bombus* spp. and Lepidoptera	Maintained higher wild bee species richness and equivalent syrphid and butterfly richness over six years.	Austria	[84]
Semi-natural elements (SNE)	*Agricultural landscapes*	*Bombus* spp. and wild bees	Increased wild bee richness, abundance and functional diversity, whereas agricultural intensification reduced them.	Austria	[156]
Artificial field defects	*Oilseed rape*	Lepidoptera, *A. mellifera, Bombus* spp., wild bees, wasps and Syrphidae	Enhanced bee, wasp and butterfly abundance and species richness, while hoverflies remained unaffected.	Czech Republic	[43]
Planted flower plots	*Agricultural landscapes*	*A. mellifera, Bombus* spp., wild bees, wasps, Diptera, Lepidoptera and Coleoptera	Increased pollinator abundance and wild bee richness compared with control plots.	Canada	[96]
Refugia flowering plants	*Mango*	Syrphidae, Lepidoptera, Apidae and other Diptera	Increase of the presence of pollinators and natural enemies	Indonesia	[36]
Prairie strips	*Maize and soybean*	Wild bees	Prairie strips enhanced plant–pollinator network complexity compared with field margins.	USA	[50]
	Maize and soybean	*A. mellifera*, wild bees and *Bombus* spp.	Enhanced bee abundance, richness and diversity across landscapes with varying complexity.	USA	[47]
Mid-field strip	*Maize*	Wild bees, *Bombus* spp., *A. mellifera* and Syrphidae	Supported higher wild bee abundance and diversity through complementary early-season floral resources.	France	[145]
Natural forest, hedgerows and grassland	*Blueberry*	*A. mellifera, Bombus* spp., wild bees, Syrphidae, Lepidoptera and Bibionidae	Forest cover increased wild pollinator abundance and functional-group richness, whereas hedgerow and grassland cover had negative effects, depending on cultivar.	Argentina	[159]
Forest edge, grassland and wildflower strips	*Sunflowers*	*A. mellifera, Bombus* spp., wild bees and Syrphidae	Forest edges and grasslands supported higher pollinator abundance and richness than wildflower strips and sunflower fields.	Serbia	[151]
Inter-row vegetation	*Vineyards*	Wild bees and Syrphidae	Both sown and spontaneous inter-row vegetation supported higher wild bee and hoverfly abundance than tilled inter-rows.	France	[107]
	Vineyards	*A. mellifera*, wild bees and Syrphidae	Higher insect richness and abundance were consistently observed across vineyards, with responses varying over time.	Spain	[117]
Cover crops	*Hazelnut*	*A. mellifera,* wild bees and Syrphidae	Native perennial cover crops supported higher pollinator abundance and diversity than native annual and conventional cover crops.	USA	[124]
	Corn and soybean	*Bombus* spp. and wild bees	Increased wild bee richness and abundance, while red clover supported bumble bees.	USA	[98]
	Peach, nectarine and plum	*A. mellifera*, *Bombus terrestris*, wild bees, Syrphidae, Diptera and butterflies	Greater pollinator benefits were observed under high management intensity.	Spain	[48]
Herb cover	*Olive*	Wild bees, Syrphidae, other Diptera, Coleoptera, non-bee Hymenoptera and Lepidoptera	Low-intensity herb cover management and complex landscapes promoted more diverse and less specialised plant–pollinator interaction networks.	Spain	[166]
Flowering areas	*Wheat*	Wild bees, Syrphidae and butterflies	Enhanced the abundance of wild bees, butterflies and hoverflies, and increased pollinator species richness.	Italy	[35]
Orchard Edges and Floral Enhancement Plots	*Citrus*	*Bombus* spp., *A. mellifera,* wild bees, Syrphidae, Lepidoptera and Coleoptera	Floral enhancements increased local pollinator abundance and diversity, but orchard-level responses were region-dependent.	USA	[34]

Table S2. Distribution of pollinator response directions to agroecological infrastructures across the studies included in the review, according to pollinator group. Values represent the percentage of group-specific classifications reporting exclusively positive, neutral or negative responses, or combinations of response directions. Each study contributed one classification for each pollinator group assessed and could therefore contribute to more than one pollinator group. Percentages were calculated separately within each pollinator group.

Pollinator group	Main effect						
	Positive (%)	Neutral (%)	Negative (%)	Neutral + Negative (%)	Positive + Negative (%)	Positive + Neutral (%)	Positive + Negative + Neutral (%)
**Bees**	54,5	9,1				36,4	
**Bumble bees**	53,5	15,5	7,0		4,2	18,3	1,4
**Coleoptera**	35,3	35,3			5,9	23,5	
**Diptera**	31,6	36,8	5,3		5,3	5,3	15,8
**Honey bees**	41,9	21,6	6,8	1,4	8,1	14,9	5,4
**Hymenoptera**						50,0	50,0
**Lepidoptera**	46,2	17,9	0,0		12,8	20,5	2,6
**Other pollinators**			50,0			50,0	
**Syrphidae**	49,3	11,6	5,8		7,2	21,7	4,3
**Wasps**	50,0	20,0	15,0		5,0	10,0	
**Wild bees**	53,4	6,9	9,5		7,8	17,2	5,2

Table S3. Distribution of pollinator response directions to agroecological infrastructures across the studies included in the review, according to the main agroecological infrastructure category. Values represent the percentage of group-specific classifications reporting exclusively positive, neutral or negative responses, or combinations of response directions. Each study contributed one classification for each pollinator group assessed and could therefore contribute multiple group-specific classifications within the same infrastructure category. Percentages were calculated separately within each infrastructure category.

Main agroecological infrastructure category	Positive (%)	Neutral (%)	Negative (%)	Neutral + Negative (%)	Positive + Negative (%)	Positive + Neutral (%)	Positive + Negative + Neutral (%)
Floral resource enhancement	41,0	16,3	10,7	0,6	11,8	13,5	6,2
Semi-natural herbaceous habitat enhancement	47,4	23,7	3,1		1,0	19,6	5,2
Woody habitat enhancement	70,8	16,7	4,2			4,2	4,2
Habitat enhancement schemes	61,5	5,1	2,6			30,8	
Within-field vegetation enhancement	65,5	3,4				31,0	
Multiple strategies	43,8	12,3	8,2		12,3	20,5	2,7
